# Strategies for *Aedes* mosquito control: A review of national guidelines from selected countries in Asia and Oceania

**DOI:** 10.1016/j.onehlt.2026.101359

**Published:** 2026-02-11

**Authors:** Seyed Aria Nejadghaderi, Rasoul Ebrahimi, Mohammad Khalili, AliAkbar Haghdoost, Abbas Aghaei-Afshar, Hamid Sharifi

**Affiliations:** a HIV/STI Surveillance Research Center, and WHO Collaborating Center for HIV Surveillance, Institute for Futures Studies in Health, Kerman University of Medical Sciences, Kerman, Iran; b Knowledge Hub for Migrant and Refugee Health, Institute for Futures Studies in Health, Kerman University of Medical Sciences, Kerman, Iran; c School of Medicine, Shahid Beheshti University of Medical Sciences, Tehran, Iran; d Department of Pathobiology, Faculty of Veterinary Medicine, Shahid Bahonar University of Kerman, Kerman, Iran; e Modeling in Health Research Center, Institute for Futures Studies in Health, Kerman University of Medical Sciences, Kerman, Iran; f Research Center of Tropical and Infectious Diseases, Kerman University of Medical Sciences, Kerman, Iran; g Institute for Global Health Sciences, University of California, San Francisco, San Francisco, CA, USA

**Keywords:** Dengue fever, National guidelines, *Aedes* control, World Health Organization, Integrated vector management, Public health

## Abstract

Dengue remains a major public health challenge, and national strategies for *Aedes* control vary widely across countries. This review synthesizes official national guidelines from 14 countries across Asia, the Middle East, and the Pacific to identify shared priorities and key differences. Most guidelines emphasize integrated vector management frameworks, combining environmental, chemical, and biological measures, alongside strong community engagement and structured surveillance systems. Several countries incorporate digital tools, adult mosquito monitoring, or innovative approaches such as Wolbachia-based biocontrol. Despite these strengths, gaps persist, including limited adoption of adult surveillance, over-reliance on chemical control, and variable coordination across sectors. Understanding these cross-country patterns may support more coherent, evidence-informed policy development for long-term and sustainable *Aedes* control.

## Highlights

• Integrated Vector Management is central to *Aedes* mosquito control in countries.
• Surveillance systems, supported by rapid diagnostics, are prioritized for dengue outbreak prevention.
• Community engagement and public education are critical for vector control efforts.
• Innovations like Wolbachia biocontrol and eco-friendly insecticides address insecticide resistance.
• Challenges include over-reliance on chemical methods, resource limitations, and fragmented inter-agency coordination.

## Graphical abstract

This figure shows the multi-faceted approach of IVM, which combines environmental, chemical, and biological interventions to mitigate the proliferation of *Aedes* mosquitoes and reduce dengue transmission. Data-driven decisions guide prevention and control measures, including environmental management (e.g., source reduction and habitat modification), chemical control (e.g., eco-friendly insecticides), and biological control (e.g., Wolbachia-based biocontrol). Community engagement and public education play a role in fostering ownership and participation in prevention efforts through awareness campaigns and volunteer training. In the event of outbreaks, rapid response protocols and contingency plans ensure timely diagnosis, treatment, and insecticide deployment. Cross-sector collaboration among health, agricultural, environmental, and municipal sectors optimizes resource allocation and strengthens implementation.

**Figure:**
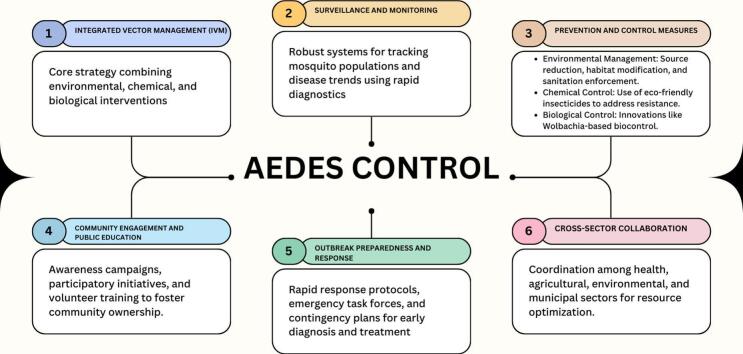
Control measures for *Aedes* mosquitos.

## 1 Introduction

*Aedes* mosquitoes are key vectors for several arboviruses, including dengue, Zika, yellow fever, and chikungunya, causing significant global health burdens. The primary dengue vectors are *Aedes aegypti* and *Aedes albopictus*, which exhibit distinct ecological and behavioral traits that necessitate tailored control strategies. *Aedes aegypti* is highly anthropophilic and urban-adapted, preferentially breeding in artificial domestic containers and resting indoors on surfaces like curtains, furniture, and walls. In contrast, *Aedes albopictus* is more exophilic and versatile, thriving in peri-urban or rural environments by breeding in natural or semi-natural sites and resting primarily outdoors in vegetation. These differences extend to insecticide susceptibility, with *Aedes aegypti* often showing higher resistance in urban areas due to repeated exposure, while *Aedes albopictus* may require alternative approaches like biological control or habitat modification. Generalizing control measures without species differentiation can limit effectiveness, as it may overlook *Ae. albopictus*'s outdoor preferences or lead to suboptimal resource allocation in regions where both species co-occur (1).

Dengue fever (DF) has become particularly concerning, spreading from nine countries in the 1970s to 128 today (2). First reported in 1779 in Jakarta (3), DF now represents a significant public health challenge with high morbidity and mortality rates. Effective clinical management can reduce mortality to below 1% (4). The World Health Organization (WHO) noted a dramatic surge in dengue cases, exceeding 5 million in 2019, with approximately 96 million people experiencing symptoms annually [2], [5]. In 2023, more than six million cases and over 6000 deaths were reported across 92 countries (6). This alarming increase and dengue's expansion to new regions prompted the WHO to declare a health emergency appeal in 2024 (7).

Dengue virus (DENV) is a neurotropic virus capable of infecting central nervous system cells, leading to potential neural damage during acute infections. This damage may arise from direct viral invasion or through antibody-dependent enhancement mechanisms (8). In addition to these direct pathways, severe systemic dengue can also produce neurological complications indirectly, leading to encephalopathy driven by metabolic disturbances, acute liver or kidney dysfunction, shock, or systemic hemorrhaging [9], [10], [11]. The typical incubation period for DENV is about seven days, during which individuals may experience headaches, fever, muscle pain, joint discomfort, and skin rashes. Dengue, known as “breakbone fever,” is classified under the flavivirus genus and is characterized as a positive-sense single-stranded RNA virus with a protein capsid and an envelope (12).

The epidemiology of key *Aedes*-borne arboviruses, such as dengue, Zika, and chikungunya, reveals the inherent limitations of sector-specific control measures. These pathogens circulate in complex transmission cycles that intrinsically link human populations, animal reservoirs, including non-human primates in sylvatic cycles (e.g., for dengue and yellow fever) and potentially livestock in certain rural-interface settings, and the environmental conditions that support vector proliferation. Consequently, achieving effective and sustainable control demands an integrated approach (13).

The One Health framework, which recognizes the fundamental interconnectedness of human, animal, and environmental health, provides the essential conceptual and operational paradigm for addressing such multifaceted challenges. It advocates for coordinated, cross-sectoral surveillance, joint risk assessment, and integrated intervention strategies. This approach is critical given the ecological determinants that shape *Aedes* ecology and transmission dynamics, such as urbanization patterns, climate variability, water-storage practices, waste-management systems, and land-use changes. These factors simultaneously influence human exposure, animal reservoir habitats, and mosquito breeding sites, underscoring the need for strategies that bridge public health, veterinary, and environmental sectors (14).

Therefore, this review systematically compares national *Aedes* control guidelines through an explicit One Health lens. We aimed to evaluate the extent to which these policies operationalize this holistic framework by integrating veterinary and environmental perspectives into traditionally public health-centric programs. Specifically, guidelines from selected countries in Asia and Oceania, including Taiwan, India, Oman, Singapore, Malaysia, Sri Lanka, Indonesia, Pakistan, the United Arab Emirates (UAE), China, the Philippines, Japan, and Australia, were assessed not only for their public-health components but also for their environmental-management strategies, mechanisms for inter-agency coordination (e.g., with veterinary and municipal bodies), and the inclusion of climate-responsive measures. By analyzing strategies across diverse epidemiological, environmental, and socio-economic contexts, this review aimed to provide an integrated overview of current approaches and identify pathways toward more comprehensive, One Health-aligned national strategies for controlling Aedes mosquitoes and the diseases they transmit.

## 2 Methods

To identify relevant national guidelines, we conducted a structured search across official governmental websites, national public health portals, and WHO regional repositories. For each included country, we reviewed publicly available documents issued by ministries of health, national disease control programs, and regulatory agencies. Supplementary searches were performed in Google Scholar and WHO databases using terms such as “*Aedes* control guideline”, “national dengue guideline”, and “vector control policy” combined with each country's name. Only official national documents available in English or with accessible translation were included, and when multiple versions existed, the most recent update was selected.

**Fig. 1:**
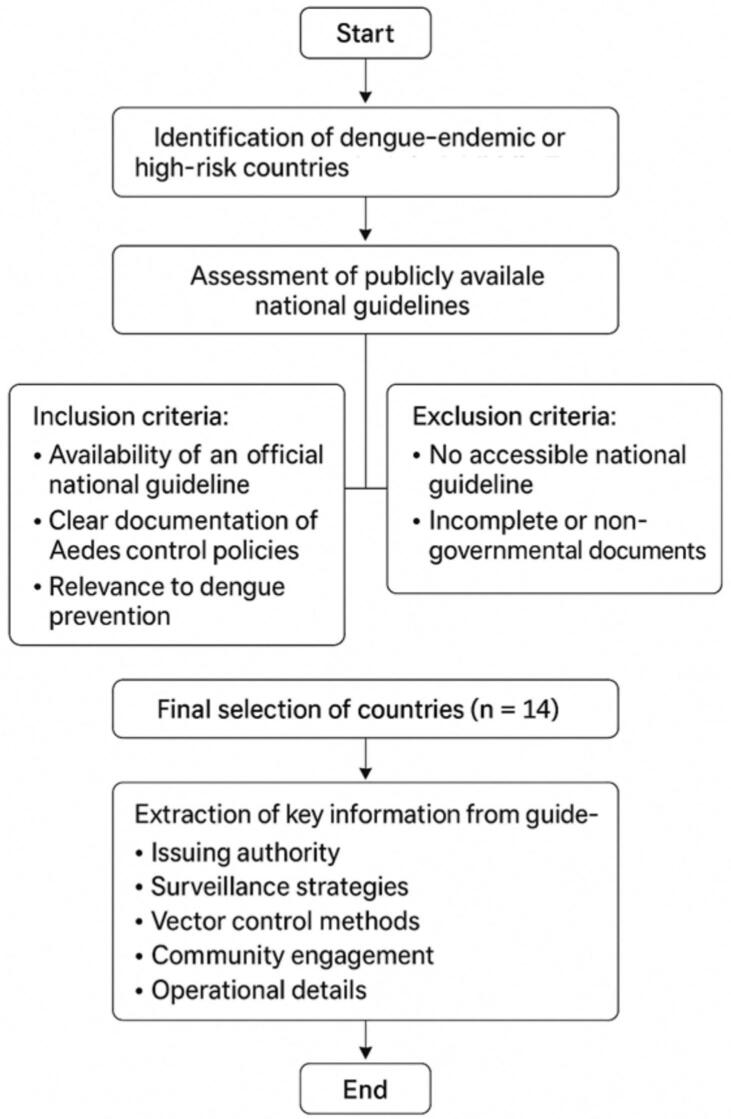
Flow chart of the country selection and guideline extraction process.

We mostly focused on countries from Asia and the Middle East due to their high dengue burden, availability of detailed national guidelines, and regional diversity in control strategies. We reviewed national guidelines in Taiwan, Iran, India, Oman, Singapore, Malaysia, Sri Lanka, Indonesia, Pakistan, the UAE, China, the Philippines, Japan, and Australia for preventing and controlling DF and *Aedes* mosquitoes. We selected countries for this review based on clear criteria to provide a representative analysis. They were selected based on the availability of national guidelines, high dengue incidence, and regional relevance to the study's focus on diverse yet comparable public health systems. Priority was given to countries with a high dengue burden and established national control programs with accessible official guidelines on *Aedes* mosquito control. We also considered geographic diversity and differences in socio-economic and healthcare systems to capture varied approaches. Countries without publicly available or detailed guidelines were excluded. International sources were reviewed to gather relevant information on this topic. Initially, the criteria for selecting relevant guidelines and protocols were defined. This study extracted all existing guidelines in the Ministries of Health or pertinent ministries of the selected countries regarding controlling *Aedes* mosquitoes and transmitting diseases, such as DF. After identifying the relevant guidelines, key information was analyzed. The key information extracted from each guideline included: issuing authority, surveillance strategies, vector control methods, community engagement efforts, and operational details. We analyzed and compared key elements of these guidelines, focusing on the clarity, coverage, and alignment of each component with WHO recommendations and Integrated Vector Management (IVM) principles. We systematically extracted data from each national guideline, noting the issuing authority, year of publication, and update status when available. These guidelines were comparatively examined in the next phase to identify their strengths and weaknesses. Fig. 1 summarizes the country selection process, inclusion and exclusion criteria, and the steps undertaken for guideline identification and data extraction.

### 2.1 Policy review framework

To systematically evaluate and compare each national guideline, we adopted a structured review framework comprising six key domains: (1) Governance and Coordination, (2) Surveillance Strategies, (3) Vector Control Interventions, (4) Community Engagement and Education, (5) Implementation and Operationalization, and (6) Monitoring and Evaluation.

### 2.2 Governance and coordination

We examined how each guideline defines leadership structures (e.g., which ministry or agency oversees *Aedes* control), intersectoral partnerships (e.g., collaboration with environment or agriculture sectors), and legal/regulatory mechanisms that support vector control efforts.

### 2.3 Surveillance strategies

We recorded whether guidelines recommend larval and/or adult mosquito surveillance, describe data integration (e.g., linking entomological and epidemiological information), and specify thresholds or indicators (e.g., Breteau index cutoffs) for triggering control actions.

### 2.4 Vector control interventions

We cataloged recommended methods (environmental/source reduction, chemical larviciding/adulticiding, biological controls such as Bti or Wolbachia, and outbreak-focused space spraying or indoor residual spraying [IRS]). We noted any guidance on insecticide resistance management.

### 2.5 Community engagement and education

We assessed how each guideline incorporates community mobilization or public education campaigns, including whether it provides details on communication channels, school-based programs, or involvement of local volunteers.

### 2.6 Implementation and operationalization

We reviewed the practical guidance regarding staffing (e.g., entomology teams, health workers), logistics (e.g., supply chains for insecticides), training requirements, and budget/funding recommendations.

### 2.7 Monitoring and evaluation

We evaluated whether guidelines set clear performance metrics (e.g., reduction targets for *Aedes* density), recommend interval assessments, or describe mechanisms for reviewing and updating strategies (e.g., periodic guideline revisions).

### 2.8 Quantitative synthesis of guideline elements

To provide a more objective comparison across national guidelines, we performed a presence/absence tally of six pre-specified core elements for each included national guideline. Two reviewers (SAN and RE) independently extracted data and recorded whether each element was explicitly described in the guideline (present = 1; absent = 0). Discrepancies were resolved by discussion and consensus with a third author (HS) when needed. We report simple descriptive summaries as counts and percentages. Where useful, narrative comparisons complement these summaries; we avoided causal language and subjective terms not supported by the extracted data.

## 3 Results

In order to better quantify trends across national guidelines, we tallied the frequency of certain core elements. Of the 14 national guidelines reviewed, 12 explicitly referenced community participation as a strategy, and 11 incorporated the WHO-endorsed IVM approach. Larval surveillance was included in all 14 guidelines, while adult mosquito surveillance was mentioned in only five. Seven countries specified thresholds (e.g., Breteau Index) to trigger vector control interventions, and six provided guidance on managing insecticide resistance (Table 1).

**Table 1:** Details of national dengue control guidelines reviewed. This table lists the issuing authority, publication year, latest update (if available), and references for each guideline included in the review. It provides transparency about the versions analyzed and recognizes that guidelines may change over time.

Country	Issuing authority	Year	Reference
Taiwan	Taiwan Centers for Disease Control (CDC)	2014	(15)
Iran	Ministry of Health and Medical Education	2022	[16], [17]
India	National Vector Borne Disease Control Program	2023	(18)
Oman	Ministry of Health	2023	(19)
Singapore	National Environment Agency (NEA)	2024	(20)
Malaysia	Ministry of Health	2023	(21)
Sri Lanka	Ministry of Health	2019	(22)
Indonesia	Ministry of Health	2021	(23)
Pakistan	Ministry of Health	2012	(24)
United Arab Emirates	Abu Dhabi Quality and Conformity Council	2021	(25)
China	National Health Commission	2014	(26)
Philippines	Ministry of Health	2024	(27)
Japan	Japan MHLW	2014	(28)
Australia	Australian Government Department of Health	2020	(29)

Abbreviations: CDC: Centers for Disease Control, NEA: National Environment Agency, MHLW: Ministry of Health, Labour and Welfare.

### 3.1 Structured appraisal of national guidelines

Using the six-domain framework described above, we systematically reviewed 14 national *Aedes* control guidelines. Table 2 summarizes each country's approach across the domains of governance and coordination, surveillance strategies, vector control interventions, community engagement and education, implementation and operationalization, and monitoring and evaluation. In the Appendix, we present our findings for each country organized by these domains, which allows for direct comparison of strengths, gaps, and unique features among different national programs.

**Table 2:** Comparative summary of national dengue control guidelines and strategies across selected countries.

Country	Surveillance Type (Larval / Adult)	Data Integration (Entomological, Epidemiological, Clinical)	Control Measures (Chemical, Biological, Environmental)	Vector Control Centralization (Centralized / Decentralized)	Reference Documents	Reported Disease Burden	Stakeholder Engagement (Government, Private, Community)
Taiwan	Larval, Adult	Partial Integration	Chemical, Environmental, Public Education	Centralized	Taiwan CDC	High	Government, Community
Iran	Larval	Limited	Environmental, Educational, Reactive Operations	Centralized	Ministry of Health	Medium	Government, Universities, Community
India	Larval	Moderate	Chemical, Environmental, Educational	Decentralized	NVBDCP	High	Government, NGOs, Community
Oman	Larval	Moderate	Chemical, Environmental, Educational	Centralized	Ministry of Health	Low	Government, Community
Singapore	Larval, Adult	Full Integration	Chemical, Biological (Wolbachia), Environmental	Centralized	NEA	High	Government, Private, Community
Malaysia	Larval	Moderate	Chemical, Environmental, Educational	Centralized	Ministry of Health	High	Government, Community
Sri Lanka	Larval	Limited	Chemical, Environmental, Educational	Centralized	Ministry of Health	Medium	Government, Community
Indonesia	Larval	Moderate	Chemical, Environmental, Educational	Decentralized	Ministry of Health	High	Government, NGOs, Community
Pakistan	Larval	Limited	Chemical, Environmental, Educational	Decentralized	Ministry of Health	High	Government, Community
United Arab Emirates	Larval	Moderate	Chemical, Environmental, Educational	Centralized	Ministry of Health	Low	Government, Private, Community
China	Larval	Moderate	Chemical, Environmental, Educational	Centralized	Ministry of Health	High	Government, Community
Philippines	Larval	Limited	Chemical, Environmental, Educational	Decentralized	Ministry of Health	High	Government, Community
Japan	Larval	Moderate	Chemical, Environmental, Educational	Centralized	Ministry of Health	Low	Government, Community
Australia	Larval, Adult	Full Integration	Chemical, Environmental, Educational	Decentralized	Ministry of Health	Low	Government, Community

Abbreviations: CDC: Centers for Disease Control, NEA: National Environment Agency, NVBDCP: National Vector Borne Disease Control Program, NGO: non-governmental organization.

Global strategies for *Aedes* mosquito control emphasize integrated, multi-sectoral approaches to mitigate disease transmission and vector proliferation. Central to these efforts is the adoption of IVM, which combines environmental, chemical, and biological interventions guided by evidence-based decision-making and community engagement. Countries prioritize robust surveillance systems to monitor mosquito populations and disease trends, often enhanced by technologies such as geographic information systems for targeted responses. In addition to larval surveillance, adult mosquito surveillance is strongly encouraged. Integration of entomological surveillance data with epidemiological and clinical surveillance systems is essential for effective vector control and timely outbreak response. Cross-sector collaboration among health, agricultural, environmental, and municipal sectors optimizes resource allocation, while legislative frameworks enforce accountability through penalties for non-compliance with sanitation standards. Public education campaigns and participatory initiatives, such as environmental clean-ups and volunteer training, improve community ownership of prevention efforts. Environmental management, including source reduction and habitat modification, is widely implemented to eliminate breeding sites, complemented by innovations such as Wolbachia-based biocontrol and eco-friendly insecticides to address resistance. Outbreak preparedness relies on rapid response protocols, emergency task forces, and contingency plans for insecticide deployment and case management. Capacity-building programs improve technical readiness among healthcare workers and volunteers, while international partnerships align national guidelines with WHO standards. As summarized in Table 2, governance structures also varied: centralized approaches (e.g., Singapore, Oman, Malaysia, Iran, and China) emphasized regulatory enforcement and standardized protocols, while decentralized systems (e.g., India, Indonesia, Pakistan, and the Philippines) tended to rely more heavily on community participation and non-governmental organization (NGO) involvement. They illustrate both common foundations, such as universal reliance on larval surveillance and strong emphasis on community mobilization, and important gaps, particularly in the adoption of adult surveillance and insecticide resistance management.

### 3.2 Frequency of core elements

Across the 14 national guidelines reviewed, community engagement and education were included in 12 (85.7%) and an IVM or IVM-aligned framework was explicitly referenced in 11 (78.6%), while all 14 (100.0%) incorporated larval surveillance as a core activity; in contrast, only five (35.7%) described formal adult mosquito surveillance methods such as BG-Sentinel or Gravitraps, seven (50.0%) specified predefined entomological thresholds like Breteau index cutoffs to trigger interventions, and six (42.9%) provided explicit guidance on insecticide resistance monitoring or resistance management (Table 2).

### 3.3 Surveillance type (larval / adult)

Larval surveillance showed up in every single national guideline we looked at (14/14, 100%), and most of the time it was framed around household or container checks with the classic indices like Breteau, House, or Container serving as the main benchmarks, so for countries such as India, Indonesia, Pakistan, the Philippines, Sri Lanka, Iran, Malaysia, Oman, and the UAE, routine larval surveys basically form the backbone of their entomological monitoring systems.

In about half of the guidelines (7/14, 50.0%) those larval indices were not just reported descriptively but were linked to predefined thresholds that would trigger stepped-up interventions, for instance, Breteau index cutoffs are explicitly written into the policies in India, Sri Lanka, and Malaysia, whereas the rest just presented the indices without clear numerical triggers, and this heavy reliance on larval monitoring really underlines how it remains the cheapest, simplest, and most deeply ingrained approach for Aedes control even though everyone knows it's not the best predictor of dengue risk.

In contrast, only five countries, Taiwan, Singapore, China, Japan, and Australia, spelled out routine adult surveillance (5/14, 35.7%), and these were the places that described more advanced systems like ovitraps, BG-Sentinel traps, or gravid *Aedes* traps that were often plugged into broader data platforms; Taiwan and Singapore, for example, both talked about large-scale ovitrap networks feeding into GIS dashboards, China has a centralized e-reporting system that integrates adult catches, Japan adds seasonal adult trapping to its national vector program, and Australia connects adult counts directly to outbreak preparedness, while the rest of the countries either mentioned adult monitoring vaguely as pilot projects or skipped it altogether.

What stands out is a pretty clear geographic and governance split: adult surveillance is mainly in countries with strong centralized systems and the resources to run them (Taiwan, Singapore, China), whereas those with more decentralized setups and tighter budgets like India, Indonesia, Pakistan, and the Philippines lean almost entirely on larval indices and community inspections, so the choice to adopt adult monitoring seems less about whether it is useful and more about whether the system has the infrastructure, funding, and national coordination to pull it off (Table 2).

### 3.4 Data integration (entomological, epidemiological, clinical)

When it comes to integrating entomological, epidemiological, and clinical data, the national guidelines showed quite a bit of variation. Only about half of the countries (7/14, 50.0%) described a system where entomological indices were explicitly linked to epidemiological surveillance and case reporting, and an even smaller group included integration with clinical or laboratory-confirmed data. Countries with strong centralized systems, Taiwan, Singapore, China, Malaysia, and Oman, tended to report the most advanced integration, often using digital dashboards or GIS-enabled platforms that combine larval indices, adult trap data, and dengue case counts to guide weekly or seasonal interventions. For example, Taiwan and Singapore both described centralized GIS dashboards where entomological data flow directly into decision-making systems that are also fed by case notifications, while China reported a national electronic reporting platform that connects adult surveillance results with epidemiological and clinical indicators.

In contrast, guidelines from more decentralized systems such as India, Indonesia, Pakistan, and the Philippines often kept entomological data streams separate from epidemiological or clinical surveillance, relying instead on local reporting mechanisms without a standardized national framework for integration. These documents mentioned routine larval surveys and case reporting but rarely detailed how the two were combined for decision-making, which suggests that local authorities may carry out integration informally but without national protocols or shared platforms. A few countries, such as Australia and Japan, reported partial integration, for example, adult mosquito monitoring or case clusters were linked regionally, but the connection to clinical data was not clearly standardized at the national level (Table 2).

### 3.5 Control measures (chemical, biological, environmental)

All 14 national guidelines emphasized environmental management as the backbone of *Aedes* control, with universal recommendations for source reduction through community clean-up campaigns, container elimination, and improved water storage practices. Countries such as India, Indonesia, Pakistan, the Philippines, and Sri Lanka described large-scale community mobilization drives coordinated with municipal services, while more centralized systems like Singapore, Taiwan, Malaysia, and China coupled environmental clean-up with strict enforcement mechanisms, including legal penalties for non-compliance. This universal reliance on environmental management reflects its long-standing role as the most sustainable and cost-effective approach to reducing *Aedes* breeding sites.

Chemical interventions were also widely included, with 12 of 14 guidelines (85.7%) referencing larviciding and adulticiding. Temephos and Bti (*Bacillus thuringiensis* israelensis) were the most commonly cited larvicides, while adult control often involved thermal fogging or space spraying during outbreaks. Countries like Malaysia, Sri Lanka, and the Philippines recommended larvicide use as a routine measure, while Singapore and Oman emphasized chemical control as a reactive outbreak strategy tightly regulated by national protocols. Only Australia and Japan placed comparatively less emphasis on chemical interventions, reflecting both different ecological contexts and more reliance on environmental or biological approaches.

Biological and innovative measures were less consistently reported, with six of 14 countries (42.9%) mentioning pilot or complementary strategies. Wolbachia-infected *Aedes* releases were documented in Taiwan, Singapore, and Australia, while Bti was included in several guidelines as a biological larvicide. Some countries, such as Indonesia and Malaysia, noted interest in ecological or biological control but described these as research trials rather than routine operational components (Table 2).

### 3.6 Vector control centralization (centralized / decentralized)

Out of the 14 national guidelines we reviewed, nine (64.3%) described a centralized model where a single national authority takes the lead in dengue vector control, sets technical standards, and coordinates implementation across different levels of government. Countries in this category included Taiwan, Singapore, Malaysia, China, Oman, Iran, Sri Lanka, the UAE, and Japan, and what stood out in their documents was the strong emphasis on uniform protocols, national dashboards, and in some cases legal enforcement for example, Singapore and Taiwan both detailed systems where central agencies deploy standardized traps, oversee data collection, and enforce penalties for non-compliance. However, Malaysia and China described national guidelines that tightly regulate larviciding and outbreak response procedures. This top-down model generally provides more consistency and ensures that new tools like GIS-linked dashboards or Wolbachia pilots are rolled out under structured oversight, though it also requires heavy investment in centralized infrastructure.

By contrast, five countries (35.7%), India, Indonesia, Pakistan, the Philippines, and Australia, reflected a decentralized approach, where responsibility is pushed down to state or municipal authorities, with the national body acting more as a coordinator or technical advisor. In these countries, local health departments or municipal governments often organize community clean-up campaigns, manage larvicide distribution, and adapt strategies to local environmental conditions, while NGOs and community health workers play a much more visible role. For instance, India's guidelines highlight the role of Accredited Social Health Activists at the village level, and the Philippines describe barangay-based inspection and reporting teams as a backbone of implementation. This structure allows flexibility and responsiveness to local contexts but can also lead to heterogeneity in execution and make it harder to enforce uniform standards nationwide.

When comparing the two models, it is clear that centralized systems lean on standardization, regulatory enforcement, and technology-driven integration, whereas decentralized systems rely more on community mobilization, local adaptation, and partnerships with NGOs or local government units. Both approaches align with WHO's IVM framework in different ways, centralization supports evidence-based decision-making through national data platforms, while decentralization strengthens community participation and local ownership, and the diversity across countries shows how governance models are shaped not just by epidemiological needs but also by political structures, resource availability, and the scale of the health system (Table 2).

### 3.7 Reference documents

Across the 14 national guidelines, there was variation in how reference materials and supporting documents were presented, some countries published highly detailed, regularly updated guidelines with clear citations to WHO recommendations and regional policy frameworks, while others relied on shorter circulars, ministerial decrees, or operational manuals with limited referencing. Countries with strong centralized programs such as Singapore, Taiwan, Malaysia, and China typically released comprehensive, standalone guideline documents that cite WHO's IVM framework and regional technical reports, often accompanied by appendices or standard operating procedures for field teams. Oman, Iran, Sri Lanka, and the UAE also provided structured national manuals, though these were sometimes less frequently updated or not as extensively cross-referenced with WHO materials.

In contrast, countries with more decentralized structures, for example India, Indonesia, Pakistan, and the Philippines, often described national guidance in the form of shorter policy documents, notifications, or strategy papers that delegate substantial operational details to state or municipal authorities; in these cases, references were limited and sometimes implicit, with WHO guidance mentioned but not systematically integrated into the text. Australia and Japan, while high-resource settings, leaned more on regional or state-level documents and professional society guidelines rather than a single consolidated national manual, reflecting their decentralized health governance systems.

### 3.8 Reported disease burden

Out of the 14 national guidelines we assessed, most came from countries with a high dengue burden. This group includes Taiwan, India, Singapore, Malaysia, Indonesia, Pakistan, China, and the Philippines, that is eight out of 14 (57.1%). In these countries dengue is basically endemic, and outbreaks show up all the time, often in seasonal waves. For example, Singapore and Malaysia have well-established national action plans because they deal with recurring epidemics. India, Indonesia, and the Philippines report high case numbers year after year, across multiple regions. Meanwhile, Taiwan, China, and Pakistan also face regular outbreaks that sometimes even spill into neighboring areas.

Two countries, Iran and Sri Lanka, fall into the medium burden group (14.3%). In Iran, the number of cases is still relatively low, but the risk is growing due to imported infections and favorable ecological conditions in the southern provinces. Sri Lanka has seen major epidemics over the past decade, but the numbers fluctuate a lot from year to year, which is why it is considered medium.

The last four, Oman, the UAE, Japan, and Australia, are in the low burden category (28.6%). Here, dengue transmission is rare, geographically limited, or mostly imported. For instance, Japan and Australia occasionally report local outbreaks, usually linked to travelers bringing the virus back from endemic regions. Oman and the UAE have reported only limited local transmission so far, even though the climate is suitable for *Aedes* mosquitoes.

### 3.9 Stakeholder engagement (government, private, community)

All of the evaluated countries had their ministries of health or national disease control programs leading *Aedes* control. This makes sense since dengue prevention usually needs strong top-down coordination. Countries like Taiwan, Singapore, Malaysia, and China showed really centralized systems where the government takes full charge, sets the policies, and enforces rules like sanitation laws or fines for breeding sites.

On the community side, 12 out of 14 countries mentioned some type of community participation. This could be through school programs, volunteer groups, or neighborhood clean-up drives. For example, Indonesia and Sri Lanka rely heavily on trained volunteers to do household inspections and spread awareness, while the Philippines uses barangay-level health workers to engage local residents. Iran also mobilizes mosques and local volunteers, showing how cultural and social structures are used to get people involved.

When it comes to private sector engagement, it is less consistent. Only about five countries (35.7%), including Singapore, Malaysia, the UAE, Taiwan, and India, explicitly mentioned working with private companies. In Singapore, private pest control services are integrated into the national system, and construction companies are held accountable for monitoring breeding sites. In the UAE, community associations and private pest control firms join government campaigns. in other places, the private role is less formal, mostly through NGOs or local businesses supporting awareness drives.

### 3.10 Comparative synthesis

To facilitate direct comparison across national documents, Table 3 summarizes key features of each guideline. Two clear governance models emerged. Centralized systems emphasize standardized national protocols, integrated data platforms, and regulatory enforcement. However, decentralized systems rely mostly on local adaptation and community mobilization. Adult mosquito surveillance is described in a minority of guidelines and is concentrated in higher-resource or more centralized programs, whereas all reviewed guidelines include routine larval surveillance. Innovative biological approaches are documented in a subset of countries and are typically framed as pilot or complementary strategies.

**Table 3:** Key comparative features of included national *Aedes* control guidelines.

Country	Surveillance (Larval / Adult)	Adult surveillance (explicit)	Notable emphasis / innovations
Taiwan	Larval, Adult	Yes	Large ovitrap network + GIS dashboards; pilot novel tools
Iran	Larval	No	Environmental management, reactive operations
India	Larval	No	Large community mobilization; routine larval indices
Oman	Larval	No	Outbreak-focused chemical control; regulated protocols
Singapore	Larval, Adult	Yes	Wolbachia deployments, Gravitraps, strong enforcement
Malaysia	Larval	No	Integrated Vector Management-aligned guidance, emphasis on enforcement
Sri Lanka	Larval	No	Volunteer networks, community campaigns
Indonesia	Larval	No	Community/non-governmental organization-driven implementation
Pakistan	Larval	No	Limited integration; mostly larval-focused
United Arab Emirates	Larval	No	Low-burden focus; public–private engagement
China	Larval, Adult	Yes	National e-reporting; integrated entomology/epi systems
Philippines	Larval	No	Barangay-level inspections; decentralized operations
Japan	Larval, Adult	Yes	Seasonal adult trapping; localized outbreak response
Australia	Larval, Adult	Yes	Adult surveillance tied to outbreak preparedness; some Wolbachia/gravitrap pilots

## 4 Discussion

We reviewed various countries' national dengue prevention guidelines, including Taiwan, Iran, India, Oman, Singapore, Malaysia, Sri Lanka, Indonesia, Pakistan, the UAE, China, the Philippines, Japan, and Australia. The guidelines highlight the importance of integrated vector management, community engagement, and tailored public health policies. Countries with robust surveillance systems and intersectoral collaboration, such as Singapore and Malaysia.

Across the 14 national guidelines reviewed, source reduction emerged as the most consistently recommended strategy, with all 14 countries incorporating environmental control measures into their national plans. Educational or community engagement initiatives were also a major focus, explicitly included in 12 of the 14 guidelines. While most countries support chemical control methods, the specific use of larvicides such as temephos or *Bacillus thuringiensis israelensis* was not uniformly detailed across all documents, making it difficult to quantify their adoption. Insecticide susceptibility varies between species, with Ae. aegypti often showing higher urban resistance due to repeated exposure, while Ae. albopictus may require alternative biological or habitat-focused interventions. Fewer guidelines provided concrete outcome metrics. For example, Cambodia reported a 40% reduction in *Aedes aegypti* density following a six-month Bti intervention, while India cited up to 55% suppression through a combination of larviciding and environmental management. Community mobilization efforts in countries like the Philippines, Brazil, and Vietnam have been associated with declines in Breteau indices ranging from 20% to 45% over the span of a year. Indoor residual spraying and space spraying were mentioned in some country reports (e.g., India, Oman, and the Philippines), though these were typically described as supplementary or reactive measures, and detailed impact evaluations were less commonly provided.

The One Health framework, which emphasizes the interconnectedness of human, animal, and environmental health systems, plays a crucial role in shaping effective *Aedes* mosquito control strategies. While often referenced in global health policies, its operationalization within national mosquito control programs is essential for comprehensive disease prevention. In this review, we examine how One Health principles are integrated or neglected in the national guidelines of 14 countries across Asia, the Middle East, and the Pacific. A key aspect of the One Health framework is intersectoral coordination, the collaborative efforts between different sectors, such as public health, veterinary health, and environmental management. Several countries have successfully integrated coordination mechanisms that allow for more effective mosquito control. For instance, these countries have established joint task forces between ministries of health, agriculture, and the environment to ensure a multi-faceted approach to vector control, where human health, environmental management, and animal health are all considered in tandem. Another critical component is shared surveillance mechanisms, where surveillance data from human health systems, veterinary services, and environmental monitoring are merged to create a comprehensive view of *Aedes* mosquito populations and their spread. The adoption of shared surveillance systems has allowed for more timely interventions and better allocation of resources. However, not all countries have fully implemented these systems, with some relying more heavily on single-sector surveillance, leading to gaps in information and delayed responses. Finally, the concept of institutional integration is essential for ensuring that Aedes control measures are consistent and sustainable across all sectors. This includes aligning policies, regulations, and monitoring efforts at national and local levels. Institutional integration is promoted through joint training programs, coordinated response plans, and cross-sector policy frameworks. Unfortunately, in other countries, institutional silos remain, hindering the effectiveness of control measures and resulting in fragmented efforts. While the reviewed national guidelines emphasize environmental control, chemical and biological interventions, and community engagement, the One Health perspective highlights the importance of recognizing the complex and interdependent factors influencing *Aedes* mosquito populations. Incorporating these principles into national strategies not only improves control efforts but also ensures a more sustainable approach to disease prevention in the long term.

While the guidelines reflect diverse national contexts, they also reveal common patterns, as well as gaps and areas where strategies could be further strengthened. The adoption of IVM in countries such as Pakistan and Malaysia signifies an approach that combines environmental, biological, and chemical control methods. IVM programs address the root causes of vector-borne diseases through community engagement and intersectoral collaboration. They integrate entomological surveillance (e.g., larval habitat mapping and insecticide resistance monitoring) with targeted interventions such as larval source reduction, biological control agents (e.g., Mesocyclops copepods), and judicious use of insecticides [30], [31], [32]. However, many IVM frameworks generalize across *Aedes* species, which can overlook *Aedes albopictus*'s broader breeding versatility and lower insecticide susceptibility in some contexts, leading to suboptimal control in mixed-vector environments. For instance, community-driven clean-up campaigns and educational outreach have proven effective in reducing *Aedes* breeding sites, aligning with evidence from cluster-randomized trials in Mexico and Nicaragua [33], [34]. Community engagement improved local ownership and the effectiveness of initiatives, as in Indonesia and Sri Lanka. This attention to community involvement leads to more sustainable, long-lasting outcomes. However, challenges persist, including inconsistent implementation, limited funding, and reliance on reactive rather than proactive strategies (35). Suitable IVM requires sustained intersectoral coordination, engaging ministries of health, environment, education, and urban planning, to address determinants like water storage practices and waste management [30], [36]. Although dengue does not involve animal reservoirs, its transmission cycle is strongly shaped by environmental and socio-ecological conditions, positioning dengue control within the environmental dimension of One Health. Many national guidelines implicitly incorporate One Health principles through environmental sanitation, urban planning regulations, climate-based surveillance, and cross-sector collaboration with municipal, environmental, or waste management authorities. However, explicit One Health framing remains limited. Strengthening these intersectoral links, especially between health ministries, environmental agencies, and urban planning sectors, can enhance the sustainability and long-term impact of dengue prevention programs.

Strengthening capacity building and policy frameworks is critical to maintaining long-term vector control efficacy, as highlighted in the WHO's Global Vector Control Response guidelines [30], [37]. In addition to these strategies, recent findings from the cluster-randomized controlled trial conducted by Morrison et al. (38), offer compelling evidence supporting the use of spatial repellents in controlling *Aedes*-borne diseases. Their study demonstrated a significant 34.1% reduction in the incidence of arboviral infections among participants using a transfluthrin-based spatial repellent compared to placebo. This represents one of the first robust *epi*demiological validations of chemical spatial repellents as an effective public health intervention for reducing disease risk. Spatial repellents may be particularly useful against *Aedes aegypti*'s indoor biting but require evaluation for *Aedes albopictus* in outdoor settings.

Recent evidence shows that dengue control can, and increasingly does, benefit from integrative, multi-sectoral approaches that go beyond classical public health control measures. For example, a recent review demonstrated how strategies combining entomological surveillance, environmental monitoring (water management, urban sanitation, vector habitat mapping), and molecular/omics tools build a comprehensive control framework (14). Spatial-ecological analyses from regions in Brazil show that arbovirus incidence (including dengue, Zika, chikungunya) correlates not only with human case data, but also with socio-demographic and environmental variables, such as population density, urban expansion, sanitation coverage, and environmental vulnerability, highlighting the importance of integrating environmental and social determinants (39).

Systematic reviews of *Aedes*-borne arboviruses underline that predictive risk mapping becomes more accurate when environmental covariates (e.g. rainfall, land-use, temperature, urbanization patterns) and socio-economic context are included, supporting the idea that dengue transmission must be understood within a broader socio-ecological system [40], [41], [42], [43]. Based on these insights, our review interprets national dengue guidelines not only as public health directives but as embedded within a broader ecological and socio-environmental system. This framing allows discussion of how guideline components related to environmental sanitation, vector habitat management, water storage regulation, waste management, urban planning coordination, and surveillance of vector populations, in addition to case detection, may serve as operational approximations of an integrative approach. Such a holistic understanding can help policymakers and public health authorities design more sustainable, cross-sectoral dengue prevention and control programs, resilient to socio-environmental changes such as urban growth or climate variability.

Singapore's vector control strategy has evolved from relying heavily on routine insecticide application to a comprehensive integrated vector management approach. This strategy prioritizes environmental management, active community participation, and the targeted use of insecticides primarily during outbreak situations (44). Surveillance methods have also advanced, moving beyond traditional larval indices to include innovative tools such as Gravitraps for monitoring adult mosquito populations. Ho et al. (45) further emphasize Singapore's flexible and data-driven approach, which integrates source reduction, legislative measures, and risk-based surveillance. Although dengue incidence has increased due to factors like viral diversity and low herd immunity, these adaptive strategies have contributed to lowering mosquito populations and enhancing control effectiveness.

The trial also highlighted several operational advantages of spatial repellents, including ease of deployment and community acceptance, which make them a promising complement to existing vector control measures. Importantly, the protective effects were sustained over the study period, suggesting that spatial repellents could offer continuous protection in endemic settings.

Establishing dengue control programs with sustained funding across all endemic countries is crucial to transition from reactive control efforts to more proactive surveillance and prevention strategies, ensuring better preparedness and effective disease management. Public-private partnerships are vital in bolstering dengue control efforts. These collaborations help mobilize resources, boost community involvement, and promote sustainable vector management. By bringing together government agencies and private sector stakeholders, such partnerships address financial and logistical challenges, enhance program execution, and provide ongoing support for control measures. Emphasizing and expanding these partnerships is key to creating resilient and effective vector control systems.

Moreover, surveillance systems for monitoring vector populations and disease incidents in regions like Taiwan and the UAE allow for prompt responses to outbreaks. These systems often use advanced technologies, including remote sensing and geographic information systems, to track breeding sites. An overreliance of surveillance systems on traditional larval indices (e.g., House Index, Breteau Index) remains problematic, as these metrics poorly correlate with adult female *Aedes aegypti* densities [46], [47]. These systems often fail to detect cryptic breeding sites, such as septic tanks, storm drains, and roof gutters, which contribute significantly to adult mosquito populations but evade conventional larval surveys [46], [48]. The lack of validated density thresholds for adult mosquitoes limits actionable insights to predict transmission risks [49], [50]. Operational gaps include inconsistent integration of adult-focused tools like BG-Sentinel traps and autocidal gravid ovitraps, which capture epidemiologically relevant populations [51], [52], and underutilized pupal surveys that could identify high-productivity containers for targeted control [53], [54]. Fragmented data systems hinder efforts by failing to synthesize entomological metrics with real-time climate or human behavioral data [55], [56]. To address these gaps, experts recommend prioritizing adult mosquito surveillance, validating region-specific transmission thresholds through longitudinal studies linking trap data to disease incidence [57], [58], and enhancing geographic information system platforms to integrate multi-source data for dynamic risk mapping and precision resource allocation [56], [59].

The over-reliance on chemical insecticides in countries like India and Singapore risks accelerating insecticide resistance and harming non-target species, underscoring the urgent need for sustainable alternatives. In contrast, Singapore's adoption of Wolbachia-based biocontrol and Gravitrap systems highlights the promise of biological and ecological interventions. The release of the wAlbB strain [60], [61], [62], [63] leverages immune priming by activating the Toll and JAK/STAT pathways in *Aedes aegypti*, upregulating antimicrobial peptides (e.g., cecropins, defensins) and reactive oxygen species to suppress DENV replication [64], [65], [66]. Concurrently, resource competition between Wolbachia and DENV for host lipids and cholesterol further restricts viral propagation [67], [68], [69]. This dual mechanism—immune activation and metabolic interference—resulted in a 71–88% reduction in DF cases in Singapore (70). Complementing this, Gravitrap systems disrupt mosquito breeding by targeting larval habitats, reducing vector density through ecological means. This integration of biological and ecological strategies is similar to approaches used in Brazil, where Wolbachia deployments were associated with a 38% reduction in dengue incidence [71], [72], and in Yogyakarta, Indonesia, where transmission dropped by 77% with an 83% decline in severe cases [73], [74]. The synergy between Wolbachia-mediated pathogen blocking (via immune priming and lipid competition) and Gravitrap-driven vector suppression minimizes reliance on chemical insecticides, aligning with sustainable vector control frameworks [75], [76], [77].

Guidelines from Iran and the Philippines frequently lack coordination between the health and environmental sectors, undermining effective management of breeding habitats. This lack of coordination can result in conflicting priorities and inefficient use of resources. Dependencies on multiple organizations, particularly in Iran, may reduce coordinated responses, causing delays. Moreover, inadequate funding in countries like Sri Lanka and Indonesia threatens the operational capacity of mosquito control strategies. Limited financial resources impede the implementation of comprehensive vector control programs.

In comparison, national guidelines and control strategies from the Americas, particularly Brazil, show both similarities and differences with those from Asia and Oceania. For instance, Brazil's national program for dengue control (Programa Nacional de Controle da Dengue) integrates epidemiological surveillance, vector control (mechanical, biological and chemical), environmental sanitation, health-education, community mobilization and intersectoral coordination, similar in structure to guidelines from Asia and Oceania (78). Empirical evidence from Brazil, however, shows that standard measures alone have had limited long-term success. In this regard, a field evaluation in Boa Vista found that despite extensive household-level vector-control visits, larvicide and insecticide applications, and water-container management, the reduction in *Aedes* density was modest and did not translate into sustained suppression of dengue incidence (79).

While the guidelines from Asia and Oceania reveal diverse governance models and capacities, examining the case of Brazil has insights into the operational challenges of implementing integrated, One Health-aligned strategies in practice. Brazil's Programa Nacional de Controle da Dengue formally integrates epidemiological surveillance, vector control (environmental, chemical, and biological), sanitation, health education, community mobilization, and intersectoral coordination, resembling the comprehensive frameworks seen in countries like Singapore and Malaysia. The program operates within the decentralized structure of the Sistema Único de Saúde, which guarantees universal health access and integrates surveillance actions across federal, state, and municipal levels. This decentralized model shares similarities with systems in India, Indonesia, and the Philippines, where local authorities hold significant implementation responsibility. However, Brazil's experience underscores how decentralization can both enable and hinder integration. While municipal health teams have flexibility to adapt interventions to local conditions, fragmentation often arises in coordinating environmental sanitation, urban planning, and vector control, particularly in resource-constrained municipalities. This mirrors challenges seen in decentralized Asian systems, where intersectoral coordination remains uneven despite national policy directives.

Empirical evidence from field evaluations, such as in Boa Vista, demonstrates a persistent gap between policy design and sustained impact. Despite regular household visits, larvicide applications, and container management, reductions in *Aedes* density were often modest and did not translate into durable decreases in dengue incidence. This discrepancy underscores a challenge highlighted in our review. The difficulty of moving from multi-sectoral policy documents to functionally integrated, locally-adapted implementation. A key feature of Brazil's approach is the integration of epidemiological and environmental surveillance through the Notifiable Diseases Information System and the Environmental Surveillance Information System. In theory, this allows for real-time data sharing between health and environmental sectors, supporting targeted interventions. In practice, however, operational silos persist, and data integration is often incomplete. This gap shows even in countries with advanced digital platforms (e.g., China, Singapore), the functional linkage between entomological, epidemiological, and environmental data streams remains a work in progress.

Community engagement in Brazil is institutionalized through endemic disease control agents (Agentes de Combate às Endemias), who conduct household visits, promote source reduction, and link communities with municipal health services. This cadre of community-based personnel is comparable to volunteer networks in Indonesia and Sri Lanka, and resembles the Accredited Social Health Activists in India. The program exemplifies how national guidelines can be operationalized through local human resources, fostering community ownership. Yet, its effectiveness is constrained by high turnover, limited training, and inadequate supervision, challenges that also plague community-based systems in Asia.

Brazil's recent adoption of innovative tools, such as Wolbachia-infected mosquito releases in Rio de Janeiro and Niterói, demonstrates how high-burden countries are piloting biological interventions within national policy frameworks, which led to significant reductions in dengue incidence align with successes in Singapore and Australia, suggesting that embedding such innovations into national guidelines requires not only technical capacity but also sustained funding, regulatory adaptability, and community trust. The Brazilian example, therefore, reinforces a finding from our cross-country analysis. The effectiveness of national guidelines depends less on the mere inclusion of One Health principles and more on the existence of operational mechanisms for intersectoral collaboration, continuous monitoring, adaptive management, and meaningful community participation in the face of ecological and socioeconomic complexity.

The surge in DF cases over the past 50 years is due to increased urbanization, migration, inconsistent water supply, and climate change, which has expanded the geographical range of *Aedes aegypti*, the main mosquito vector for dengue [80], [81]. *Aedes* mosquitoes breed in artificial containers, thriving in densely populated urban environments with stagnant water, such as in discarded tires or construction sites [82], [83]. In addition, human responses to water scarcity can affect mosquito habitats. Individuals often store water around their homes in regions lacking suitable water sources, particularly during droughts [84], [85]. This disrupted water supply pattern has been linked to DF outbreaks in Cuba and Brazil, and other mosquito-borne diseases in India (82). These water storage practices may become more prevalent as climate change progresses due to rising temperatures and diminishing rainfall (83).

*Aedes aegypti* eggs resist desiccation, allowing them to endure extended drought periods and complicating control efforts (86). Main control strategies include applying insecticides through indoor residual spraying and using skin repellents (87). However, these approaches often prove ineffective or unsustainable. While these methods may lower mosquito populations, there is limited evidence that they significantly impact disease incidence (31). Growing vector resistance to insecticides and environmental contamination pose additional challenges to effective insecticide use (87). A systematic review of vector control and DF prevention highlighted a significant lack of global data for evaluating intervention programs (31). Furthermore, the transmission of mosquito-borne diseases, including DF, is influenced by environmental factors like temperature, rainfall, and humidity [85], [88], [89]. Temperature affects mosquito vector physiology, behavior, and DENV development [86], [90]. Mechanistic models suggest optimal transmission occurs at 29 °C for *Aedes aegypti* and 26 °C for *Aedes albopictus* (90). However, statistical models have produced varying results. Studies in Thailand indicated DF transmission peaks at temperatures of 30 °C or higher, while research in Taiwan found months with average temperatures exceeding 18 °C associated with increased DF rates. Vezzani et al. showed that temperatures above 20.8 °C were conducive to *Aedes aegypti* population growth (83). Seasonal DF patterns are partially driven by rainfall, which creates stagnant water sources near human habitats. While humid conditions accompany rainfall, high humidity alone can prevent mosquito egg desiccation. Hales et al. identified average annual vapor pressure as the most significant predictor of DF distribution (91). A study in Taiwan highlighted that favorable weather conditions could facilitate the transition of imported DF cases into local epidemics (92). Also, in various regions, the incidence of dengue fever DF is linked to vegetation indices, tree cover, and land use, as these environmental factors influence the population size of mosquito vectors. Adult *Aedes aegypti* are more commonly found in areas with constructed buildings and medium-height trees (81). The interplay of rainfall, temperature, and humidity with land cover creates microclimates, which can lead to variability within urban areas that are conducive to *Aedes* mosquito habitation (81).

Looking across the 14 national guidelines, it is obvious that geography shapes the scale and style of dengue control. Tropical and subtropical countries like Malaysia, Indonesia, Singapore, India, Sri Lanka, and the Philippines, struggle with year-round transmission or intense seasonal peaks. Their hot, humid climates plus monsoon rains create perfect breeding grounds for *Aedes* mosquitoes, so their guidelines push continuous surveillance and community-driven clean-up campaigns. On the flip side, more temperate countries such as Japan and Australia only see outbreaks in specific regions (Southern Japan, Northern Queensland), and usually during the warmer months. Here, dengue is not endemic everywhere, so control strategies are more localized and tied to outbreak preparedness rather than constant year-round activity.

Environmental conditions make the differences even clearer. In the Middle East countries (e.g., Oman, the UAE, Iran), rapid urbanization, high construction activity, and water storage practices are the biggest risks. *Aedes* love stagnant water at construction sites or stored in tanks during dry months. So, the guidelines there lean heavily on urban planning rules, sanitation standards, and community awareness about container management. In southeast Asia, rainfall and flooding are the key drivers, which is why Malaysia, Indonesia, and the Philippines combine seasonal vector campaigns with school- and community-based education. China and Taiwan stand out because they have linked climate data, GIS, and disease surveillance into one system, allowing them to forecast when and where breeding peaks will happen.

Then there is the socioeconomic side, which might be the most important for understanding trends. High-income countries like Singapore, Taiwan, Australia, and Japan can invest in high-tech solutions (Wolbachia-based biocontrol, gravitraps, drone-based habitat mapping) and back them up with strong legislation and enforcement. For instance, Singapore fines property owners if breeding sites are found, which ensures accountability. In middle-income countries such as India, Indonesia, and the Philippines, resources are more limited, so they rely on mobilizing huge networks of volunteers, community health workers, and NGOs to sustain interventions. It is cost-effective but can be patchy and less standardized. In lower-resource settings like Pakistan or Sri Lanka, budgets are often insufficient for proactive measures, so responses lean more on chemical interventions once outbreaks start, instead of consistent long-term strategies.

Together, the big picture is that geography decides whether dengue is endemic or seasonal, environmental conditions define the main risk factors and breeding habitats, and socioeconomic capacity determines how advanced or community-driven the response can be. High-burden tropical countries with stronger economies (like Singapore and Malaysia) focus on integrated, multi-sectoral management with sustainable funding. Countries with weaker resources but still high transmission (like Sri Lanka or Pakistan) rely more on reactive measures, often struggling to keep up during outbreaks. Low-burden countries (Oman, the UAE, Japan, Australia) prioritize preparedness, strict regulation, and targeted responses to imported or localized cases.

While dengue is primarily a human-mosquito-human transmission cycle without a recognized animal reservoir, a comprehensive One Health approach remains relevant for integrated vector management and eco-epidemiological surveillance. Recent literature underscores the potential role of animal health systems and veterinary public health in strengthening dengue preparedness and response, even in the absence of classical zoonotic spillover. For instance, Gwee et al. (93) systematically reviewed evidence of dengue virus detection in diverse animal species, including non-human primates, bats, and domestic animals, highlighting the possibility of enzootic transmission or spillback events that could influence viral circulation dynamics. Although acute infection in animals appears limited, such findings suggest that animal surveillance could serve as an early warning system for viral activity, particularly in peri-urban and rural settings where human-animal-vector interfaces are prominent. Furthermore, veterinary entomology and animal health services can contribute to coordinated vector surveillance and control. The French Agency for Food, Environmental and Occupational Health and Safety developed a One Health evaluation framework for integrated vector management systems, emphasizing multisectoral collaboration between public health, veterinary, and environmental sectors (94). This approach highlights how veterinary expertise in entomological monitoring, resistance management, and habitat assessment can augment national dengue control programs. In many of the reviewed guidelines, however, explicit linkages with veterinary authorities or animal health surveillance were rarely described. This gap may limit the ecological breadth of vector control strategies.

Climate and environmental determinants of dengue transmission further reinforce the need for intersectoral coordination. Abdullah et al. (95) demonstrated through meta-analysis how climate variables such as precipitation, temperature, and humidity correlate with dengue incidence, factors that also influence vector breeding sites in both human and animal environments. Veterinary services often monitor climate-sensitive diseases and could support integrated data platforms that combine entomological, epidemiological, and environmental data, a synergy aligned with the One Health emphasis on holistic eco-epidemiological monitoring.

From a governance perspective, several national guidelines reviewed (e.g., India, Indonesia, Pakistan) operate under decentralized structures where community and NGO engagement is prominent. Incorporating animal health stakeholders, such as livestock departments, wildlife agencies, or veterinary associations, into these existing networks could enhance habitat management, especially in agricultural or peri-urban areas where *Aedes* breeding sites overlap with animal holdings. Moreover, veterinary vaccine research and biotechnology advances, as noted by Procopio et al. (14), offer parallel insights for dengue vaccine development and vector control innovations, such as Wolbachia-based biocontrol, which itself emerged from entomological and microbiological research bridging human and insect health.

Although several national guidelines incorporate environmental sanitation and inter-ministerial coordination, elements often associated with One Health, few articulate structured governance mechanisms that integrate human, animal, and environmental health sectors with clear accountability and data-sharing protocols. In contemporary One Health scholarship, the framework is understood not merely as intersectoral collaboration but as a systemic approach with defined institutional roles, shared indicators, and integrated surveillance across domains.

In practice, most national programs operate under health-sector leadership with ad hoc involvement of municipal or environmental agencies, lacking formal mandates for veterinary services or wildlife health authorities. This limits opportunities for leveraging veterinary entomological capacity, animal disease surveillance networks, or zoo-prophylactic sentinel systems that could enhance early detection of arbovirus activity. Furthermore, data systems remain largely siloed. Entomological indices, human case reports, climate data, and animal health observations are seldom integrated into a unified platform for real-time risk assessment. Without shared digital infrastructure and agreed-upon metrics, One Health remains aspirational rather than operational.

To move beyond conceptual mentions, future guidelines should specify: Governance structures that include representation from veterinary, agriculture, environment, and urban planning sectors in vector control decision-making bodies; joint monitoring frameworks with common indicators (e.g., vector density, animal seropositivity, climate thresholds) that trigger coordinated responses; unified data-sharing platforms that link human, animal, and environmental data streams, supported by clear data governance policies; cross-sectoral training and capacity building to foster a shared understanding of One Health objectives among field staff. Such institutionalization of One Health principles would align with global frameworks like the Tripartite (WHO-OIE-FAO) collaboration on zoonoses and enhance the sustainability and effectiveness of *Aedes* control programs, even for non-zoonotic arboviruses like dengue.

While this review synthesizes structural and strategic elements of national *Aedes* control guidelines, a critical examination reveals persistent assumptions that may limit their effectiveness. First, the widespread reliance on larval surveillance and container indices rests on the assumption that controlling immature stages translates to reduced adult populations and disease transmission. However, evidence indicates that larval indices often poorly correlate with adult female density and dengue risk, particularly in settings with cryptic breeding sites such as septic tanks and storm drains. This over-reliance may stem from historical precedent, lower cost, and operational simplicity, yet it may also reflect a gap in technical capacity and funding for adult surveillance systems.

Second, the predominant focus on *Aedes aegypti* in many guidelines, often without explicit differentiation from *Aedes albopictus*, assumes that control measures effective for one species will suffice for both. This oversight neglects ecological and behavioral differences, such as *Aedes albopictus*'s exophilic tendencies and broader habitat range, potentially undermining control efficacy in regions where both species co-exist.

Institutional and operational barriers further constrain the implementation of integrated approaches. Despite frequent mention of Intersectoral collaboration, guidelines often lack concrete mechanisms for sustained coordination between health, environmental, municipal, and planning sectors. For example, in decentralized systems like India and Indonesia, local adaptations are flexible but often suffer from fragmented data sharing and inconsistent resource allocation. Conversely, centralized systems like Singapore's enforce standardization but may struggle with community ownership and adaptability at the local level.

The integration of One Health principles remains largely implicit in most guidelines. While environmental management, climate adaptation, and urban planning are acknowledged, few documents explicitly frame these actions within a holistic One Health framework that connects human, animal, and environmental health governance. This gap may stem from siloed ministerial mandates, competing budgetary priorities, and the absence of mandated cross-sectoral committees with decision-making authority. Moreover, monitoring and evaluation frameworks rarely assess the ecological or social determinants of transmission, focusing instead on entomological or case-based outcomes.

To move beyond descriptive summaries, future guideline revisions should explicitly address species-specific strategies for *Aedes aegypti* and *Aedes albopictus*, incorporate adult surveillance indicators and validate threshold-based triggers, establish formal, resourced intersectoral committees with clear mandates, integrate climate data and land-use patterns into routine surveillance platforms, and adopt outcome metrics that measure cross-sectoral collaboration and environmental impact. Without confronting these underlying assumptions and structural barriers, national guidelines risk perpetuating reactive, siloed, and ecologically narrow approaches to *Aedes* control.

Our study showed a gap between the rhetorical endorsement of One Health and its tangible operationalization in most national *Aedes* control guidelines. To bridge this gap, we propose an actionable framework derived from the synthesis of existing strengths and identified weaknesses. The first step involves establishing formal joint governance structures, such as permanent national and sub-national One Health Task Forces for Arboviral Diseases. These bodies should be co-chaired by public health, veterinary services, and environmental protection authorities, and must operate with a clear mandate and dedicated budget to ensure sustained cross-sectoral leadership. Concurrently, implementing unified digital data platforms is essential to aggregate human clinical data, veterinary laboratory reports, including those on unexplained animal deaths or primate surveillance, and entomological indices in real-time. Such integration enables the generation of early warning signals based on a holistic view of the transmission ecosystem.

Moving from surveillance to action, the development of cross-sectoral intervention protocols is paramount to move beyond siloed responses. In practice, this means that during an outbreak in peri-urban or rural zones, coordinated protocols would synchronize public health teams conducting fever surveillance and household vector control, veterinary teams sampling livestock and wildlife in adjacent areas while advising farmers, and municipal environmental teams managing irrigation canals, parks, and waste disposal sites. Furthermore, animal health must be integrated into long-term strategic planning. This entails prioritizing research on the role of domestic and peri-domestic animals in local transmission cycles and evaluating the potential use of veterinary vaccines for reservoir species in high-risk ecological niches as part of a comprehensive control strategy. Finally, the framework's sustainability hinges on capacity building and community engagement. Field officers from all relevant sectors require training on basic One Health principles for arboviruses, while community awareness campaigns should educate the public not only about eliminating mosquito breeding sites but also about reporting sick animals and participating in integrated surveillance efforts. By adopting such a structured, operational framework, national *Aedes* control programs can evolve from fragmented efforts into cohesive, resilient, and more effective strategies that address the true complexity of arboviral disease ecosystems.

### 4.1 Strengths and limitations

Our study has some limitations. First, the guidelines reviewed are restricted to specific countries and regions, and any future shifts in disease outbreak patterns could influence the reliability of these results. We primarily focused on national guidelines for managing and controlling *Aedes* mosquitoes, which may not cover all pertinent strategies within broader vector control efforts. However, by including countries with high dengue incidence and mortality rates, the findings provide greater generalizability across different regions. Moreover, this review does not apply a standardized success metric, as national guidelines vary in context, structure, and availability of outcome data. Observations are based on documented strategies rather than implementation outcomes. It is also important to note that while this review included a diverse set of countries across Asia and the Middle East, it did not cover other dengue-endemic regions such as South America and sub-Saharan Africa. This was mainly due to limited public access to official national guidelines from those regions or language constraints that made detailed comparison difficult. As a result, the geographic scope of our analysis may not fully capture the global variation in *Aedes* mosquito control strategies. Future studies should aim to include countries from Latin America and Africa, where dengue continues to pose a serious public health challenge, to provide a more comprehensive and globally representative assessment of national policies. Also, some guidelines may not have been found, which does not imply that they have not been used by the countries. By synthesizing information from various nations, this study highlights adaptable strategies that can be tailored to various socio-cultural contexts. It is also important to note that the presence or absence of specific elements in our tallies reflects what was explicitly documented in the available national guidelines rather than the full scope of activities implemented in practice. Some countries may carry out adult surveillance, insecticide resistance monitoring, or community-based interventions even if these strategies were not formally included or clearly described in their published guidelines. As such, the apparent absence of guidance in a national document should not be interpreted as definitive evidence that the activity is not performed. This limitation underscores the challenges of relying solely on document-based policy reviews and highlights the need for future research to complement guideline analyses with operational assessments and field-level data. Additionally, many guidelines do not explicitly differentiate between *Aedes aegypti* and *Aedes albopictus*, leading to potential limitations such as reduced control efficacy in areas with both species, where strategies may favor *Aedes aegypti*'s urban focus and overlook *Aedes albopictus*'s outdoor breeding and lower insecticide susceptibility.

## 5 Conclusions

National guidelines for *Aedes* mosquito control demonstrate a range of approaches aimed at reducing the burden of vector-borne diseases. Many incorporate community engagement, IVM, and surveillance systems as core components. These elements are encouraged in international guidance and may help support more responsive and sustainable programs. However, challenges such as dependence on chemical control, limited coordination across sectors, and constrained resources are still reported in several contexts. Addressing species-specific differences between *Aedes aegypti* and *Aedes albopictus* remains a main gap, as generalizations can limit tailored interventions. Strengthening collaboration between agencies and securing long-term funding remain key priorities for advancing effective vector control efforts.

## Human ethics and consent to participate declarations

Not applicable.

## CRediT authorship contribution statement

**Seyed Aria Nejadghaderi:** Writing – review & editing, Writing – original draft, Visualization, Validation, Software, Resources, Methodology, Investigation, Data curation, Conceptualization. **Rasoul Ebrahimi:** Writing – review & editing, Writing – original draft, Methodology, Data curation. **Mohammad Khalili:** Writing – review & editing, Writing – original draft, Supervision, Methodology, Conceptualization. **AliAkbar Haghdoost:** Writing – review & editing, Writing – original draft, Validation, Supervision, Project administration, Methodology, Investigation, Conceptualization. **Abbas Aghaei-Afshar:** Writing – review & editing, Writing – original draft, Validation, Resources, Investigation. **Hamid Sharifi:** Writing – review & editing, Writing – original draft, Validation, Supervision, Resources, Project administration, Investigation, Data curation, Conceptualization.

## Consent for publication

Not applicable.

## Ethics approval and consent to participate

The ethics committee of Kerman University of Medical Sciences, Kerman, Iran approved the present study protocol (ethics code: IR.KMU.REC.1403.232).

## Funding

None.

## Declaration of competing interest

The authors declare that they have no known competing financial interests or personal relationships that could have appeared to influence the work reported in this paper.

## Data Availability

No data was used for the research described in the article.

## Appendix A Supplementary data

Supplementary material: An overview of national dengue prevention and control frameworks across selected countries Image 1
