# Supplementary material for: Strategies for Aedes mosquito control: A review of national guidelines from selected countries in Asia and Oceania
Source: One Health. 2026 Feb 11;22:101359. doi: 10.1016/j.onehlt.2026.101359 (PMC12925428; doi:10.1016/j.onehlt.2026.101359)
Supplement: Supplementary file 1 — Supplementary material: An overview of national dengue prevention and control frameworks across selected countries [file mmc1.docx]

**Appendix.**

**Taiwan Centers for Disease Control and Prevention**

**Governance and Coordination**

The Taiwan Centers for Disease Control (CDC) leads national dengue control, working closely with municipal health bureaus and local governments. During outbreaks, an Incident Command System brings together public health, environmental, and academic stakeholders for coordinated decision-making. Legal mandates impose fines on premises violating sanitation standards, reinforcing a multi-sector accountability framework.

**Surveillance Strategies**

Both larval and adult surveillance are mandated. Ovitraps are distributed to households to collect eggs, while BG-Sentinel or gravid *Aedes* traps capture adult females. All data feed into a centralized GIS‐enabled dashboard linking entomological findings with clinical case reports, allowing weekly updates during peak season to guide targeted interventions.

**Vector Control Interventions**

Environmental management—community‐led clean‐up events and strict enforcement of container removal—is primary. Chemical control involves temephos larviciding and, as needed, thermal fogging for adulticiding. Wolbachia‐infected male releases have been piloted in select municipalities to reduce population fitness. Indoor residual spraying is invoked in high-incidence districts but remains secondary to source reduction.

**Community Engagement and Education**

A sustained public education campaign runs via multimedia, school programs, and volunteer training. Residents report mosquito sightings through hotlines and mobile apps, creating a feedback loop that strengthens two‐way communication. Neighborhood leaders organize volunteer inspection teams to identify and eliminate breeding sites proactively.

**Implementation and Operationalization**

Local health bureaus manage procurement of larvicides and equipment under CDC guidelines. Rapid Response Teams stand ready to investigate clusters and deploy control packages—mosquito nets, larvicide, and educational materials—particularly in high-risk districts. Field personnel receive standardized, pre-season training on chemical and biological control methods to promote swift, uniform actions.

**Monitoring and Evaluation**

Entomological indices (Breteau, Container, House indices) and epidemiological data (case counts, outbreak detection times) are reviewed weekly during transmission season. Post-season evaluations inform guideline updates, incorporating insights from university research and local health bureaus. Thresholds for intervention (e.g., specific Breteau index cutoffs) are predefined and trigger automated response protocols.

**Iran**

**Governance and Coordination**

The Ministry of Health and Medical Education spearheads Iran’s dengue response, supported by a Permanent Defense Committee Against Biological Threats that convenes representatives from multiple ministries (Agriculture, Defense, Environmental Protection) and institutes (Pasteur Institute). Municipal governments manage urban sanitation, while the Inspector General’s Office enforces compliance with biological defense regulations. This inter-agency structure is formalized through operational plans and dedicated budgets for biological threat mitigation.

**Surveillance Strategies**

Iran’s 2021 protocol mandates integrated surveillance of both human cases and mosquito populations. Provincial health centers conduct routine larval surveys and feed data into a centralized Disease Management Center. Entomological metrics (e.g., Breteau index) are tracked alongside clinical case reports, though actual linkage varies by province depending on local reporting capacity. The Meteorological Organization supplies climate data to predict mosquito breeding peaks, informing timing of field activities.

**Vector Control Interventions**

Environmental management is prioritized: municipalities lead clean-up campaigns to remove or modify breeding habitats, bolstered by legal provisions (e.g., Article 688) that expedite waste management improvements. Larviciding and localized fumigation are implemented reactively following confirmed cases. While the guidelines reference WHO-recommended larvicides, they do not specify chemicals by name. The Pasteur Institute conducts insecticide resistance testing to guide operational choices and studies *Aedes* ecology to refine intervention targeting.

**Community Engagement and Education**

Public awareness is driven by the Iran Broadcasting Organization, supplemented by school‐ and mosque‐based campaigns. Trained volunteers carry out door‐to‐door visits, distributing educational materials and demonstrating source elimination techniques. During outbreaks, provincial Rapid Response Teams hold community meetings to reinforce hygiene practices. However, the protocol does not define standardized indicators for measuring the effectiveness of these campaigns.

**Implementation and Operationalization**

Operational responsibilities are divided among provincial health centers, municipalities, and relevant ministries. The Ministry of Agriculture oversees quarantine measures for animal and plant threats, while the Environmental Protection Organization manages pollution reduction efforts. Hospitals and clinics are directed to improve case management capacity (e.g., increasing bed availability) in parallel with vector control operations. The guideline calls for interdisciplinary training exercises and allocates budgets for joint preparedness drills, but it does not specify detailed timetables for each activity.

**Monitoring and Evaluation**

Performance is assessed through monthly reviews of entomological indices (e.g., larval container index) at health management unit meetings. Outcomes feed into annual guideline revisions, informed by partnerships with academic institutions. Although climate‐informed predictive models guide planning, no explicit percentage targets for mosquito suppression are provided. The focus remains on trend analysis—observing increases or decreases over time—and adapting field tactics accordingly .

**India**

**Governance and Coordination**

India’s dengue prevention efforts are led by the National Vector Borne Disease Control Programme (NVBDCP) under the Ministry of Health and Family Welfare. The NVBDCP identifies *Aedes aegypti* as the primary vector of concern and coordinates dengue-focused control activities across central, state, and district levels. The program works in tandem with the National Health Mission to support infrastructure, intersectoral coordination, and technical oversight of dengue interventions.

**Surveillance Strategies**

India’s national guidelines emphasize larval surveillance targeting *Aedes* breeding habitats—mainly water-holding containers in residential areas and public spaces. Field teams from state and district health departments conduct surveillance. Although entomological data are routinely collected, real-time integration with clinical dengue case data is limited, which may delay targeted responses to the outbreak.

**Vector Control Interventions**

The control strategy centers on *Aedes*-specific interventions such as source reduction (elimination of breeding sites), use of larvicides like temephos, and reactive fogging in high-risk zones during dengue transmission seasons (typically pre-monsoon and monsoon periods). National guidelines also promote the evaluation and phased adoption of eco-friendly insecticides to manage insecticide resistance, in line with WHO recommendations for *Aedes* control.

**Community Engagement and Education**

Community-level engagement plays a key role in dengue prevention. Accredited Social Health Activists, community health workers, and municipal staff conduct door-to-door awareness campaigns focused on *Aedes* breeding elimination and dengue symptom recognition. Advisory committees involving local leaders and representatives support grassroots mobilization. However, standardized tools to assess the reach or impact of these campaigns are lacking.

**Implementation and Operationalization**

State and district health authorities are responsible for implementing dengue control operations, including the procurement of insecticides and fogging equipment, staff training, and logistics management. The decentralized nature of implementation allows flexibility in adapting strategies to local outbreak patterns, though comprehensive budgeting frameworks are not uniformly detailed across regions.

**Monitoring and Evaluation**

Entomological monitoring uses indices such as the Breteau index to guide interventions, with a stated goal of reducing mosquito density by 50% within six months. The NVBDCP holds annual review meetings to evaluate program effectiveness, though formal mid-course corrections or adaptive management mechanisms are not clearly outlined in the current guidelines.

**Oman**

**Governance and Coordination**

Oman’s Ministry of Health leads a national strategy that integrates environmental health and urban planning. Regional health offices adapt these guidelines locally, while multi-sector partnerships—including collaboration with Muscat Municipality.

**Surveillance Strategies**

Surveillance systems monitor both larval and adult *Aedes* populations, using geographic information systems to map breeding sites and track disease outbreaks. This real-time data supports timely interventions in high-risk areas.

**Vector Control Interventions**

Key measures include targeted insecticide applications at breeding hotspots and establishment of awareness committees. In response to resurgence events, field teams identify breeding sites for larviciding, and rapid-response “Change for Good” campaigns mobilize municipal and community actors.

**Community Engagement and Education**

Public awareness sessions and exhibitions—such as the “Change for Good” campaign—engage residents in waste-management and environmental cleanliness, fostering community ownership of vector control activities.

**Implementation and Operationalization**

A special vector-control team oversees operations, coordinating with local municipalities to manage waste and environmental hygiene. Research investments guide strategic decisions, with government-funded studies on mosquito ecology informing field approaches.

**Monitoring and Evaluation**

Oman’s framework relies on GIS-enhanced data collection to evaluate intervention outcomes. Collaborations with WHO strengthen monitoring and response capabilities, though specific entomological targets (e.g., percentage reductions) are not explicitly stated.

**Singapore**

**Governance and Coordination**

The National Environment Agency (NEA) serves as the lead authority for vector-borne disease control in Singapore, establishing overarching policies and coordinating with other governmental agencies such as the Ministry of Health and research institutes like the Environmental Health Institute. A professional working group under NEA facilitates cross‐agency communication and helps align field activities with national priorities.

**Surveillance Strategies**

Singapore’s guidelines incorporate both larval and adult mosquito surveillance. Field teams routinely inspect known breeding habitats, while Gravitraps are deployed to capture adult female *Aedes* mosquitoes and their eggs—data from these traps help assess population density in different zones. In addition, NEA explores mobile‐app–based reporting tools that enable residents to flag potential breeding sites, further enriching the surveillance dataset.

**Vector Control Interventions**

Environmental management—removal or modification of breeding habitats—is the cornerstone of Singapore’s control efforts. Complementing this, targeted chemical fogging is conducted in identified high‐risk locales. Singapore also leads in biological control innovations: the Wolbachia‐mediated Incompatible Insect Technique combined with the Sterile Insect Technique involves releasing Wolbachia‐infected male *Aedes aegypti* to induce sterility in wild females, thereby suppressing the overall population.

**Community Engagement and Education**

Public education campaigns are continuous and multifaceted; NEA leverages digital platforms, school‐based programs, and mass media to encourage residents to “keep surroundings clean.” Local volunteers—often drawn from grassroots organizations—assist with site inspections and community clean‐up drives. These efforts are designed to foster a sense of ownership among residents, creating a community‐driven extension of the national surveillance and control network.

**Implementation and Operationalization**

Implementation is underpinned by strong research infrastructure. The Environmental Health Institute conducts infection experiments (including dengue, chikungunya, Japanese encephalitis, West Nile virus, and malaria) in secure laboratories to refine intervention tools. Legislation enforces strict penalties for premises permitting breeding, and field teams receive regular training on both chemical and biological control methods. Interagency collaboration supports coordination of urban planning, environmental health, and public education activities in line with NEA’s technical guidelines.

**Monitoring and Evaluation**

Singapore integrates entomological and epidemiological data via centralized databases. Performance metrics—such as target thresholds for Gravitrap counts or Breteau indices—trigger predefined interventions. Regular evaluations allow NEA to update guidelines in response to emerging evidence (e.g., Wolbachia trial outcomes or shifts in insecticide resistance patterns), thus maintaining a dynamic, evidence‐based framework .

**Malaysia**

**Governance and Coordination**

Malaysia’s Ministry of Health leads an IVM framework, supported by legislation under the “Destruction of Disease‐Bearing Insects Act” to enforce environmental sanitation and multi‐sector collaboration. State health departments adapt national directives to local contexts, with interagency cooperation involving municipal authorities and environmental agencies.

**Surveillance Strategies**

Surveillance has shifted from exclusively residential areas to include construction sites—recognized as prolific *Aedes* breeding grounds. The Infectious Disease Control Information System centralizes larval and adult monitoring data, enabling rapid identification of emerging hotspots. This electronic system allows entomological data to flow efficiently from field teams to state and federal levels.

**Vector Control Interventions**

Environmental source reduction remains a primary strategy, complemented by chemical measures. Malaysia has transitioned from malathion to water‐based fogging formulations to combat insecticide resistance. National clean‐up campaigns (“gotong-royong”) mobilize communities to remove breeding containers, while continued research into eco-friendly larvicides informs future chemical control choices.

**Community Engagement and Education**

Community participation is galvanized through large‐scale “gotong-royong” campaigns, which bring together residents, local authorities, and NGOs to clean public spaces and educate households. Schools and media channels disseminate information on mosquito habits and preventive practices. This continuous outreach underlines the importance of shared responsibility, with community leaders often coordinating local clean‐up events.

**Implementation and Operationalization**

National IVM guidelines provide a template for state health departments to develop localized action plans. Procurement of fogging machines, larvicides, and equipment is handled at the state level, following Ministry of Health specifications. Training modules for entomology teams and field staff are updated regularly, and budget allocations for vector control operations are delineated in state health plans.

**Monitoring and Evaluation**

The National Strategic Plan for Dengue Prevention and Control sets entomological targets (e.g., acceptable *Aedes* indices) and mandates biannual assessments. State health departments report Breteau and House indices at defined intervals; data are reviewed at national IVM coordination meetings. Ongoing research—supported by the Ministry of Health—evaluates novel interventions (e.g., Wolbachia trials), ensuring that control strategies evolve in response to shifting disease dynamics, including emerging threats like Zika virus.

**Sri Lanka**

**Governance and Coordination**

The National Dengue Control Unit (NDCU), under Sri Lanka’s Ministry of Health, operates within a Presidential Task Force for Dengue Prevention and Control. Established after the 2004 outbreak, the Task Force provides high‐level oversight and promotes inter‐organizational cooperation among the Ministry, local government bodies, and partner NGOs.

**Surveillance Strategies**

Sri Lanka’s National Action Plan (2019–2023) emphasizes real‐time epidemiological surveillance and enhanced entomological monitoring. District and provincial health offices conduct larval surveys, using metrics such as the Breteau index to predict mosquito population surges. Data collection is supported by a standardized reporting framework that feeds into an electronic dashboard for national‐level analysis.

**Vector Control Interventions**

IVM is the core approach, combining source reduction, targeted larviciding, and environmental cleanup. The plan calls for intensified container control during peak transmission seasons and rapid larviciding campaigns in response to threshold breaches. Although IRS and space spraying are mentioned, they are reserved for outbreak emergencies rather than routine use.

**Community Engagement and Education**

Community involvement is structured through local “Dengue Task Forces” that conduct door‐to‐door education and coordinate clean‐up drives. Messaging is tailored to urban and rural contexts via radio, television, and social media. The 2019–2023 Action Plan specifies performance indicators—such as the proportion of households reached by education campaigns—but leaves precise evaluation methods to district offices.

**Implementation and Operationalization**

The Plan outlines roles for the NDCU in policy direction and provincial health departments in operations. A budget of 350–400 million rupees supports activities across five years, covering procurement of larvicides, training of vector officers, and public awareness materials. Field manuals are periodically updated to incorporate lessons from the 2017 outbreak, and technical assistance is provided to renovation projects (e.g., water‐storage infrastructure) to reduce breeding opportunities.

**Monitoring and Evaluation**

A performance framework with measurable indicators ((specific data points like the number of dengue cases, Breteau Index values, or coverage of awareness campaigns, used to track program performance and guide decisions) tracks incidence and mortality targets (incidence <100 cases per 100,000; mortality <1.0%). Quarterly entomological surveys measure Breteau and House indices, and results are reviewed during biannual Task Force meetings. The Plan mandates mid‐term evaluations (2021 and 2022) to recalibrate strategies based on progress and emerging urbanization challenges.

**Indonesia**

**Governance and Coordination**

Indonesia’s Ministry of Health leads a decentralized framework in which provincial and district health offices adapt national guidelines for local epidemiological conditions. Intersectoral collaboration involves municipal governments, the Indonesian Red Cross, and NGOs, all coordinated under a central task force for vector control.

**Surveillance Strategies**

Surveillance systems track mosquito populations through routine larval inspections at household and community levels; adult mosquito trapping is less emphasized. Data flow from village health posts (“Puskesmas”) to district and provincial health offices. Community‐based surveillance tools—such as those used in the December 2021 Subokerto dengue case—enable volunteers to report suspected breeding sites and suspected dengue cases, improving early detection.

**Vector Control Interventions**

IVM underpins interventions: environmental cleanup, source reduction, and targeted larviciding. In Subokerto, local volunteers used community outreach to eliminate breeding containers. The Indonesian Red Cross supports rapid response by deploying larvicide and conducting localized space spraying when clusters of cases arise.

**Community Engagement and Education**

Indonesia relies heavily on community volunteers trained by the Indonesian Red Cross. In Subokerto, volunteers visited households, educated residents on preventing stagnant water, and coordinated with local authorities to remove breeding sources. Volunteers also disseminated information at maternal and child health centers, ensuring that mothers understood preventive practices. This community‐driven model was credited with swiftly controlling the Subokerto outbreak in December 2021.

**Implementation and Operationalization**

Provincial health offices oversee procurement of larvicides and training of field personnel. Village health posts coordinate clean‐up campaigns and monitor intervention progress. The National Guidelines recommend forming multi‐disciplinary rapid response teams—including entomologists, health educators, and logistics officers—to manage outbreaks. Data from community volunteers feed into district surveillance databases, enabling timely decision‐making.

**Monitoring and Evaluation**

Indonesia’s guidelines encourage using entomological indices (e.g., Breteau index) to gauge intervention success but do not prescribe specific reduction targets. Instead, district health offices perform monthly evaluations of larval indices and case counts, reviewing outcomes at quarterly coordination meetings. The Subokerto case exemplifies this approach: volunteers’ community reports and entomological data prompted local authorities to act, and subsequent monthly surveys confirmed sustained decline in mosquito densities .

**Pakistan**

**Governance and Coordination**

Pakistan’s *Aedes* control guidelines were developed by the Malaria Control Program under the Ministry of National Health Services, Regulations, and Coordination, with stakeholder input from provincial health departments, key NGOs, and WHO country offices. A national policy framework integrates health and environmental sectors to support sustainable vector control, emphasizing coordination through provincial task forces.

**Surveillance Strategies**

Guidelines emphasize larval surveillance carried out by health department teams at district and tehsil levels. Data on container indices and larval density are collected during routine inspections and reported up through provincial offices. Although adult surveillance is mentioned as complementary, it is not systematically implemented nationwide. Clinical case data flow separately through disease notification systems, leading to limited integration with entomological findings.

**Vector Control Interventions**

The document endorses IVM in line with WHO recommendations. Core measures include environmental management (removing standing water), source reduction, and targeted larviciding (e.g., temephos application). Space spraying is reserved for outbreak response in dengue hotspots. Emphasis is placed on minimizing reliance on chemical interventions long-term by strengthening community and environmental approaches.

**Community Engagement and Education**

Community awareness campaigns are coordinated by district health authorities in collaboration with local municipalities. Educational messages—delivered via radio, print media, and community meetings—focus on eliminating breeding sites. Provincial and district-level “Dengue Task Forces” engage religious and community leaders to promote behavioral change; however, the guidelines do not specify metrics for evaluating these campaigns.

**Implementation and Operationalization**

Provincial health departments adapt national guidelines to local epidemiology and available resources. Each province is responsible for procurement of larvicides, training of field teams, and logistics management. Standard operating procedures outline roles for entomologists, health educators, and field surveillance officers. No detailed budget breakdowns are provided, and implementation timelines are left to provincial discretion.

**Monitoring and Evaluation**

Entomological indicators—primarily the Breteau index—serve as benchmarks for evaluating intervention success, with suggested thresholds for intensified action (e.g., Breteau index > 5 prompts larviciding). Outcomes are reviewed during monthly provincial health meetings, feeding into annual national reviews coordinated by the Malaria Control Program. There is no formal mid-term adjustment protocol; evaluation relies on comparisons of pre- and post-intervention indices.

**United Arab Emirates**

**Governance and Coordination**

In Abu Dhabi, the Abu Dhabi Quality and Conformity Council (QCC) leads mosquito management via a professional working group that includes municipal pest control, public health authorities, and private service providers. At the federal level, the Ministry of Health and Prevention oversees broader policy, with the National Strategic Plan for Dengue Prevention formalizing cross-sector collaboration among health, environment, and municipal agencies.

**Surveillance Strategies**

The UAE guideline endorses both larval and adult surveillance, with field teams conducting routine inspections of construction sites, public spaces, and residential compounds. Data are centralized by the QCC, which maintains a GIS-based dashboard to monitor breeding hotspots in real time. Clinical dengue case data are linked through national disease notification systems, enabling prompt outbreak detection.

**Vector Control Interventions**

Key interventions include environmental management—strict enforcement of building regulations to prevent water accumulation—source reduction, and targeted larviciding using WHO-approved chemicals. Space spraying is employed reactively during outbreaks, guided by predefined epidemiological thresholds. The plan also promotes integrated pest management, encouraging biological control options (e.g., larvivorous fish) in artificial water bodies and eco-friendly larvicides in sensitive areas.

**Community Engagement and Education**

Public education campaigns leverage mass media, school programs, and digital platforms to instruct residents on eliminating breeding sites. The QCC coordinates with community associations in each emirate to conduct “Mosquito Eradication Weeks,” during which volunteers perform inspections and distribute educational materials. Metrics for community participation—such as the number of households inspected—are tracked annually, though standardized outcome measures (e.g., reduction in container index attributable to community action) are not specified.

**Implementation and Operationalization**

Implementation relies on municipal pest control units for field operations, with technical guidance from the QCC. The UAE guideline outlines standard operating procedures for larvicide application, space spraying, and environmental cleanup. Training workshops for pest control personnel are mandated biannually. Budgeting is centralized, with federal funds allocated to emirates based on risk profiles; detailed regional breakdowns are managed by local health authorities.

**Monitoring and Evaluation**

A performance framework incorporates entomological indices (e.g., larval indices) and epidemiological metrics (case incidence rates). The QCC reviews monthly surveillance reports, triggering reactive measures when thresholds are exceeded (e.g., Breteau Index > 3). Annual evaluations assess program effectiveness, informing guideline revisions. Collaboration with WHO and regional partners supports periodic external audits, ensuring alignment with global best practices.

**China**

**Governance and Coordination**

The National Health Commission (NHC) leads China’s vector-borne disease strategy, coordinating with provincial CDCs and local health bureaus. A national steering committee convenes representatives from health, environment, agriculture, and research institutes. Provincial CDCs adapt NHC guidelines to local epidemiological contexts.

**Surveillance Strategies**

China’s guideline mandates larval and adult surveillance, with CDC field teams conducting weekly inspections of high-risk zones (e.g., tourist spots, urban neighborhoods). Ovitraps and adult traps (e.g., BG-Sentinel) are deployed systematically. Data feed into a centralized information system that links entomological surveillance with epidemiological and clinical case reporting, enabling near-real-time situational awareness.

**Vector Control Interventions**

The primary strategy is environmental management—enforcing regulations on water storage practices and promoting community clean-up campaigns. Chemical control includes temephos and pyrethroid-based larviciding, with guidance on insecticide resistance monitoring. Space spraying and IRS are reserved for outbreak containment. Biological controls, such as Bti and sterile insect releases, have been piloted in select provinces.

**Community Engagement and Education**

Public campaigns utilize television, radio, and social media to broadcast “Mosquito-Free Home” initiatives. Schools integrate dengue education into curricula. Local leaders coordinate volunteer cleanup drives, particularly before peak transmission season. Feedback mechanisms allow residents to report hotspots via a mobile app, strengthening community-government collaboration.

**Implementation and Operationalization**

Local health bureaus manage procurement of insecticides and equipment under NHC oversight. Training for field staff—covering surveillance, insecticide application, and community outreach—is conducted annually by provincial CDCs. Rapid response teams are pre-positioned in high-risk areas with stocks of larvicide, personal protective equipment, and educational materials.

**Monitoring and Evaluation**

Performance is measured via entomological indices (Breteau, Container, and House indices) and epidemiological targets (e.g., time from case detection to outbreak response). Data are reviewed weekly during dengue season by NHC and provincial CDCs. Annual national conferences evaluate outcomes and update guidelines. Partnership with academic institutions contributes to incorporating new research (e.g., climate modeling) into revisions.

**Philippines**

**Governance and Coordination**

The Department of Health (DOH) oversees national dengue prevention through the Dengue Prevention and Control Program, coordinating with local government units (LGUs) and the National Epidemiology Center. LGUs adapt DOH guidelines to their jurisdiction, with the Barangay Health Stations (BHS) serving as front-line implementation points. A National Dengue Advisory Council provides oversight and policy guidance.

**Surveillance Strategies**

The guideline emphasizes larval surveillance, conducted monthly by BHS workers and Barangay Health Volunteers, using container indices (Breteau and House indices) to guide interventions. Adult surveillance is limited, except in outbreak settings. Surveillance data flow from BHS to municipal health offices, then to regional epidemiology units, which integrate case notifications with entomological findings to inform action thresholds.

**Vector Control Interventions**

Primary control measures include source reduction via community cleanup (“4S Strategy”: Search and destroy, Self-protection, Seek early consultation, Say yes to fogging). Larviciding using temephos is applied in high-risk Barangays; space spraying is reserved for active outbreak response. The guideline also recommends pilot testing of eco-friendly larvicides and biological controls where feasible.

**Community Engagement and Education**

BHS workers and Barangay Health Volunteers implement door-to-door education, focusing on the 4S Strategy. The DOH conducts mass media campaigns—TV, radio, and social media—especially during the rainy season. Schools and religious organizations partner in awareness drives. Performance indicators include the percentage of households inspected and community participation rates in cleanup activities, though standardized impact evaluations are not specified.

**Implementation and Operationalization**

LGUs handle procurement of larvicides and fogging equipment, following DOH specifications. Training workshops for BHS staff cover surveillance techniques and public education messaging. Interagency coordination involves the DOH, Department of Environment and Natural Resources for waste management, and the Department of Education for school-based programs. Budget allocations for dengue control are part of LGU health budgets, varying by local revenue.

**Monitoring and Evaluation**

Entomological data (Breteau and House indices) are compiled monthly at BHS and transmitted to municipal health offices. Outbreak thresholds—such as a Breteau index above 5—trigger intensified interventions. The National Epidemiology Center conducts quarterly reviews of case incidence and vector indices, recommending guideline adjustments as needed. Annual program audits are conducted by DOH regional offices, incorporating feedback into the next year’s planning cycle.

**Japan**

**Governance and Coordination**

The Ministry of Health, Labour, and Welfare (MHLW) serves as the central authority for Japan’s dengue and *Aedes* control efforts, replacing the century-old “Infectious Disease Prevention Act” with the modern “Act on the Prevention of Infectious Diseases and Medical Care for Patients with Infectious Diseases.” This updated legislation emphasizes proactive public health measures, continuous monitoring, and timely information dissemination about disease risks. Provincial health departments are empowered to adapt the national guidelines to their regional contexts, coordinating with local governments, academic institutions, and community groups to implement tailored prevention programs.

**Surveillance Strategies**

Japan’s National Strategic Plan for Dengue Prevention mandates both larval and adult mosquito surveillance. Provincial teams deploy traps (e.g., ovitraps and adult‐capture traps) at high-risk sites—urban centers, ports, and tourist areas—and feed data into a centralized system that links entomological results with clinical case reports. Weekly reports during peak season inform rapid decision-making at both regional and national levels.

**Vector Control Interventions**

The national guidelines prioritize environmental management—community clean-up events, strict enforcement of container regulations, and urban planning adjustments to eliminate breeding sites. Chemical control (temephos larviciding and thermal fogging) is employed when indices exceed threshold values. During outbreaks, IRS is authorized in high-incidence districts. Pilot programs for Wolbachia-infected male releases have taken place in select municipalities to suppress *Aedes* populations.

**Community Engagement and Education**

Public awareness campaigns run year-round, with MHLW, local health bureaus, and school systems collaborating on multimedia messaging, volunteer training, and neighborhood inspections. A dedicated hotline and mobile app allow residents to report suspected breeding sites or dengue cases, creating a continuous feedback loop between communities and health authorities. Education in schools and community centers is intensified before the monsoon season.

**Implementation and Operationalization**

Local health bureaus are responsible for procurement of larvicides, training of field staff, and maintenance of trap networks. Rapid Response Teams—comprising entomologists, environmental officers, and emergency planners—are on standby to investigate suspected outbreaks within 24 hours. Pre-positioned stocks of chemicals and public health kits support rapid mobilization, and annual training workshops update personnel on the latest vector control tools and data-management software.

**Monitoring and Evaluation**

Entomological indices (Breteau, Container, and House indices) and epidemiological metrics (case incidence, time to outbreak detection) are reviewed weekly during transmission season. Post-season, MHLW convenes a national review incorporating feedback from provincial CDCs and academic partners to revise guidelines. Though specific suppression percentage targets are not stipulated, trend analyses drive adjustments in both strategy and resource allocation for the following year .

**Australia**

**Governance and Coordination**

The Australian Government Department of Health and Aged Care oversees the national dengue framework, working closely with state health departments and the Queensland Dengue Management Plan. During an outbreak, an incident management team coordinates actions across public health units, local councils, and emergency services. Governance emphasizes defined responsibilities: state agencies lead surveillance and intervention, while federal bodies provide technical guidance and funding.

**Surveillance Strategies**

Australia’s guideline mandates both larval and adult surveillance, with a particular focus on northern Queensland, where local transmission occurs annually. Field teams inspect water-holding containers, deploy BG-Sentinel and Gravid *Aedes* traps for adult monitoring, and record epidemiological data through mandatory case notifications. Real-time GIS mapping integrates entomological and clinical data, enabling rapid identification of hotspots and informing targeted responses within a 200 m radius of index cases.

**Vector Control Interventions**

Source reduction—removing or altering containers that hold water—is the foundational strategy but is labor-intensive during large outbreaks. IRS and chemical fogging (e.g., pyrethroids) are deployed when surveillance indices exceed threshold levels. Outbreaks are classified as localized or widespread, guiding whether a narrow 200 m buffer or broader area receives insecticide application. Emerging research on eco-friendly larvicides and Wolbachia trials is integrated into operational guidelines as evidence becomes available.

**Community Engagement and Education**

Public awareness campaigns emphasize “Clean Inside, Clean Outside” messaging, with schools, community groups, and local councils collaborating on cleanup drives. Residents receive information via social media, local radio, and council newsletters. Community volunteers assist with inspections and source elimination, while public-private partnerships (e.g., with tourism operators) reinforce prevention messaging among travelers.

**Implementation and Operationalization**

State health departments manage procurement of insecticides, training of field teams, and logistical support. In Queensland, Rapid Response Teams—hybrid public health and vector control units—deploy within 24 hours of a confirmed case, carrying larvicide, adulticide, educational materials, and personal protective equipment. Federal funding supports annual training workshops and maintenance of a national supply stockpile. Local councils enforce regulations on property owners to minimize breeding sites.

**Monitoring and Evaluation**

Performance metrics include reductions in entomological indices (e.g., House Index, Breteau Index) and speed of outbreak containment. Surveillance data are reviewed weekly by state public health units during peak season; post-season evaluations assess response times, cost-effectiveness, and community engagement outcomes. Findings inform the annual update of the national framework and guide investment in innovative technologies, such as drone-based larval habitat detection.
